# Platform economy: (dis-) embeddedness processes in urban spaces

**DOI:** 10.1186/s42854-021-00029-x

**Published:** 2021-12-20

**Authors:** Sina Hardaker

**Affiliations:** Department of Economic Geography, Julius-Maximilians-University Würzburg, Am Hubland, 97074 Würzburg, Germany

**Keywords:** Platform economy, Digital platform, (dis-)embeddedness, de-commodification, Accountability, Polanyi

## Abstract

Digital platforms, understood as multi-sided matchmakers, have amassed huge power, reimagining the role of consumers, producers, and even ownership. They increasingly dictate the way the economy and urban life is organized. Yet, despite their influential and far-reaching role in shaping our economic as well as sociocultural world, our understanding of their embeddedness, namely how their activities are embedded in systems of social and societal relationships and how they conceptualize their main functions and actions in relation to their wider setting, remains rudimentary. Consequently, the purpose of this frontier paper is threefold. Firstly, it reveals the need to discuss and evaluate (dis-)embedding processes in platform urbanism in order to understand the underlying dynamics of platform power and urban transformation. Secondly, it aims to reveal the main reasons in regard to the difficulties in pinpointing digital platforms embeddedness. Thirdly, it seeks to propose future research unravelling the (dis-)embeddedness of the platform economy.

This paper argues for three main reasons namely unawareness, unaccountability and non-transparency of digital platforms that drive the lack of embeddedness and reaffirms platform power. This is mainly based on the configuration of new commodities, platforms’ strategic avoidance of labour protections and other regulatory frameworks as well as platforms’ secrecy in which they operate. This frontier paper argues that transferring the concept of embeddedness to the platform economy might serve as a valuable tool to understand and pinpoint essential dynamics and relationships at play, therefore proposing embeddedness as a basis for future research on the platform economy. It strongly argues that a more detailed understanding is urgently needed, in order to be able to understand, accompany and actively influence the development of the platform economy in regulatory terms.

## Science highlights

- Identifies three main reasons for the difficulties of evaluating digital platforms (unawareness, unaccountability and non-transparency).
- Identifies inter alia the lack of embeddedness research on the commodification of ‘consumer work’.
- Highly values the embeddedness concept for analysing contemporary economic and urban transformations.

## Policy and practice recommendations

- Urban policymakers and practitioners should understand the role and power of digital platforms.
- Digital platforms form an indispensable part of urban life, controlling many urban interactions.
- The unaccountability of platforms calls for a wider regulatory framework.

## Digital platforms – reorganizing urban interactions and operations

Digital platforms, such as Alphabet, Alibaba, Amazon and Facebook, are “*programmable digital infrastructures controlled by platform operators [ …*] *who, curate the interactions of interdependent complementors and users*” (Grabher and König 2020: 104; Grabher and van Tuijl 2020: 6). They now represent some of the most valuable corporations of our times (Cabral et al. 2019), as they profit heavily from accelerating network effects (e.g. Srnicek 2017; Kenney and Zysman 2016, 2019, 2020), relatively low transaction costs (e.g. Schwarz 2017), as well as a lack of applicable regulations (e.g. Srnicek 2017; Graham 2020). Signifying “ecosystems of interaction” (Barns 2019: 3), digital platforms substantially condition and contribute to the reorganization of cities all around the world. They fundamentally transform the economic as well as social fabric of substantial aspects of our everyday lives, such as work, communication, transport and accommodation, and have paved the way for what has been coined ‘platform economy’ (Kenney and Zysman 2016, 2019, 2020). A plethora of different labels such as ‘on-demand’, ‘gig’ and ‘sharing economy’ (e.g. Richardson 2015 discusses in length the definitional difficulties of the latter) have been developed. Not surprisingly, a multitude of definitions and understandings exist, including different – sometimes even contradictory – approaches to categorizing platform models (e.g. Evans and Gawer 2016; Howcroft and Bergvall-Våreborn 2019; Kenney et al. 2019; e.g. Yablonsky, 2018; Asadullah et al., 2018; Hoffmann et al., 2021 (for B2B-platforms)). Fumagalli et al. (2018: 8), for instance, differentiate between advertising platforms (such as Google and Facebook), cloud platforms (e.g. Amazon Web Services), industrial platforms (like General Electric or Siemens), product platforms (e.g. Spotify), work platforms (like Uber, Airbnb or Deliveroo) as well as logistic platforms (such as Amazon). A large majority of these digital platforms actively shape markets, as so-called ‘market-makers’ (e.g. Schwarz 2017; Frenken et al. 2018; Kirchner and Schüßler 2020), thus engaging users “*through a participatory economic culture and mobilis [ing] code and data analytics to compose immanent infrastructures*” (Langley and Leyshon 2017: 1). Thereby, they are infiltrating a diverse range of markets. Such infiltration not only causes ‘disruption’ to established markets but also develops completely new ones, reshaping consumption practices (Bissell 2020), while also redefining value creation processes and reorganizing value chains (e.g. Schwarz 2017; Kiesling 2018; Bearson et al. 2019; Kenney and Zysman 2019; Grabher and van Tuijl 2020). Further, platforms influence and even create their institutional and regulatory framework (Frenken et al. 2018; Kirchner and Schüßler 2020), effectively becoming the market (Kenney et al. 2020) and therewith shaping our economy and society. However, this image of platforms is empirically only weakly developed (Kenney and Zysman 2020; Hardaker, 2021). Conceptualizing their ‘space’ in a wider organizational system is of importance if a more nuanced and full (er) picture of platforms and how they evolve over time is to be gained.

Consequently, this frontiers paper proposes that the concept of embeddedness, that has shaped the foundation of essential research in international business (Heidenreich 2012; Ferraris 2014), provides a suitable and helpful overall framework for future studies regarding platforms. Generally, embeddedness describes the integration of different subjects into socio-economic systems and networks (Appel 2016). While regulatory (rules, laws, regulators), normative (values, norms, categories, belief systems) and cultural-cognitive (deep, firmly anchored values) institutions form the basis for social embedding, the relationships between different actors (e.g. companies, suppliers, service providers) from social, socio-economic or organisational contexts are understood as network embeddedness. Economic geographers have consistently underlined the constantly embedded character of economic practices (Peck 2005; Cockayne 2016). Yet, they underestimate the impacts on the spatial organization of the platform economy (Wood et al. 2019; Kenney and Zysman 2020; Hardaker, 2022b), which represents a mostly under-researched aspect of urban transformation. Consequently, this paper proposes the embeddedness concept to address this blind spot, bridging the disciplinary divide between urban studies and economic geography, offering an opportunity to understand how digital platforms are (dis-)embedded in our daily social and economic life.

The paper is structured as follows. Firstly, platform urbanism and its far-reaching implications on urban transformation, is critically discussed, revealing the need to comprehend the (dis-)embedding processes in order to better understand, conceptualize and influence digital platforms evolvement and behaviour. Secondly, the paper aims to reveal the main reasons in regard to the difficulties in pinpointing digital platforms embeddedness. Consequently, this offers a possible explanation as to why the discipline of economic geography is largely neglecting platform economy and platform urbanism related discussions. Thirdly, it seeks to propose future research unravelling the (dis-)embeddedness of the platform economy.

## Platform urbanism and urban transformation

The growing influence and pervasiveness of platforms has led to numerous terms, depending on the context in which they are discussed, such as platform capitalism (e.g. Langley and Leyshon 2017), power (e.g. Culpepper and Thelen 2019), logic (e.g. Andersson 2017; Schwarz 2017) or society (e.g van Dijck et al. 2018). The increasing presence as well as power of digital platforms in urban spaces has led to the establishment of platform urbanism (e.g. Fields et al. 2020; Richardson 2020; Sadowski 2020). As digital platforms are “*woven into urban life, produce urban space, and participate in urban governance*” (Fields et al. 2020: 465), they have positioned themselves as an indispensable part of urban life in the digital age, as they “*control urban interactions*” (Graham 2020: 1) in a diverse range of urban operations, such as transport and housing (Richardson 2020: 4). Digital platforms have become “*a new significant urban governance actor* “(Aguilera et al., 2020: 19) that is not only reterritorializing existing infrastructure, but controlling critical infrastructures of urban societies (Barns 2019: 1) (see Artioli 2018 for a literature review on digital platforms and cities specifically). Prominent examples of platform-based business models causing radical market changes in cities can be found across various branches: Uber and Lyft (re-arranging transport services, e.g. Hall and Krueger 2017; Berger et al. 2018; Fan et al. 2019; Grabher and van Tuijl 2020; Walker 2020; Wells et al. 2020), Airbnb (private accommodation, e.g. Zervas et al. 2017; Aguilera et al. 2019; van Doorn 2019), Amazon (markets for goods; e.g. Culpepper and Thelen 2019; Kenney et al. 2019; Hardaker, 2021a) as well as Google, Facebook and YouTube (search, communication and social networks; e.g. van Dijck et al. 2018; Zuboff 2019; Kenney and Zysman 2020). Particularly the platforms Airbnb and Uber, both currently negatively affected by the Covid-19 crisis and the accompanying restrictions, have attracted much scholarly attention and reveal impressively how digital platforms fuel urban transformation. Cocola-Gant and Gago (2019) highlight the increasing insecurity and displacement concerns for tenants that the Airbnb business model is generating, while it simultaneously acts as an instrument that contributes to the financealization of housing. Further, they link Airbnb with gentrification. Several studies argue that Airbnb should be understood as a new urban institution that is transforming relations between market, state, and civil society actors (Ferreri and Sanyal 2018), as it inter alia assists expanding cultural commodification while simultaneously turning citizens into “place entrepreneurs” (Törnberg and Chiappini 2020). Similarly, Uber represents an “*interstitial platform infrastructure*” that depends “*on existing (often publicly funded) infrastructures like road networks, sidewalks and other public spaces ( …*)” (Stehlin et al. 2020: 5). The example of Amazon, representing a great beneficiary of the Covid-19 crisis due to governmental ordered lockdowns and accompanying accelerated online sales, reveals how a digital platform is augmenting, substituting or reorganising physical retail spaces, thereby transforming shopping streets and whole city centres (Hardaker, 2021). While retail platforms are helpful in facilitating (small) retailers’ entry to e-commerce (Battermann and Neiberger 2018) and have the potential to contribute to small brick-and-mortar retailers resilience in times of pandemic crisis due to many closed brick-and-mortar shops (Appel and Hardaker 2021), digital transaction platforms such as Amazon are heavily criticized due to their market power (Calvano and Polo 2020) and their (mis-)usage of data (Mattioli 2020). Being responsible and in control of the infrastructure that allow retailers and customers to get together, digital platforms can raise fees, change their algorithms (e.g. their recommendation algorithms to put more emphasis on price) and expect sellers to advertise if they want to maintain visibility in search results. Moreover, and particularly profitable for platforms (Hagiu et al. 2020), they are selling products on a marketplace that they simultaneously operate, thereby pushing out suppliers by directly competing with them by entering their product spaces, as in the case of Amazon (Zhu and Liu 2018; Gilbert 2020). Although digital platforms do not own the underlying assets that enable performance of the economic activity they stimulate, (for example, Uber owns no taxis, Airbnb owns no rentable real estate), they can dictate and reorganise value creation. Another clear example of the reorganisation of value creation is Google’s business model, which plays a dominant role in helping customers find merchants locally. Merchants have a strong incentive to advertise on the google platform (you do not exist, if you are not on Google), which in turn extracts value from the local market, as for example the city centres and shopping streets, and diverts it to the platform.

Generally, research agrees on the platform economy’s profound implications on several key aspects, such as the organization of urban life (Stehlin et al. 2020: 2) and socio-spatial relationships of daily workplaces (Wells et al. 2020: 3). Platform urbanism is within geography and urban studies as a means to describe the intensifying linkages between technologies and cities as well as the accompanying rising manifestation and power of the digital platforms (Sadowski 2020: 2). Yet, criticism and warnings seem to be a constant accompaniment of these studies. (Graham (2020) 3 f.) predicts that we “*end up with unaccountable and undemocratic organizations managing key digital infrastructures of our cities”* which currently represents “*an inevitable urban future of capitalism stripped down to its essentials*”. Due to their power to mediate flows of people and capital, they “*contribute [s] to the daily making and remaking of local economic geographies”* and thus, are *“undeniably embedded into the fabric of the cities in which [they] operate [s]*” (Katta et al. 2020: 2). Consequently, this paper strongly agrees with (Schwarz’s (2017) 13) argument that “*an understanding of such platform power cannot be complete unless one considers the embedded nature of digital platforms and the various dynamics at play*.” Consequently, the paper intends to evaluate in more detail the main reasons in regard to the difficulties in pinpointing digital platforms embeddedness, before discussing the usefulness of and potential research regarding the (dis-)embeddedness concept.

## Unawareness, unaccountability and non-transparency of digital platforms

(Grabher and König (2020) 93) argue that “*just like markets ( …*) *platforms are nothing natural, but are objects of ongoing political contestations that forge the embedding of the platform economy into the regulatory framework of society.*” The intellectual heritage of embeddedness lies in sociology and was coined by Polanyi (1957), who used the concept in two contradictory ways – a ‘soft’ as well as ‘hard’ Polanyi embeddedness. The ‘hard’ one describes the arising of a reactionary ‘countermovement’ whereby society attempts to re-embed the economy through the creation of social protections such as labour laws and tariffs; central to the ‘hard’ conception is the commodification of labour, the environment and money as ‘fictitious commodities’ (for a detailed differentiation see Grabher and König 2020). The ‘soft’ Polanyi has been essentially developed further by Granovetter (1985), who describes the concept as the assumption that economic activities are embedded in systems of social and societal relationships. Consequently, a company, respectively a platform conceptualizes its main functions and actions in relation to its wider setting (Pike et al. 2000). This approach to embeddedness has established a wide-ranging research agenda, especially in economic geography (Henderson et al. 2002; Hess 2004; Hess and Coe 2006; Wood et al. 2016; Burt et al. 2017). Yet, (Hess (2004)– only considering the ‘soft’ Polanyi) criticizes that an extreme emphasis on the local dimension prevents from recognizing the role of non-local embedded relationships. Consequently, Hess (2004) introduces a threefold typology by differentiating interconnected societal (cultural, institutional and historical origins of a company), network (composition and structure of network relations within and beyond the firm), and territorial embeddedness (geographical anchorage, accomplishing acceptance inter alia in regard to property and development rules as well as customers and employers). (Hess (2004) 181) emphasizes, *“[i] t is the simultaneity of societal, network and territorial embeddedness that shapes networks and the spatial-temporal structures of economic action*.” In order to achieve embeddedness, negotiation of internal and external network relationships has to take place. It also implies the adaptation to national, regional or local (e.g. legal) frameworks. Embeddedness thus describes the integration of different subjects into socio-economic systems/networks (Appel 2016). Therefore, the positions and capacities of individual subjects are placed in relation to other subjects from economic, social or territorial systems. Space is understood as a construct of different actors and forms of embedding, which form the basis for new development possibilities (transformation processes). Consequently, similar transformation processes in different places always have different outcomes, as they are determined by the existence of regional cultures and local institutions (Hess 2004).

Dissecting (Graham’s (2020): 4) statement that *“[P] latforms in the urban environment are fundamentally reshaping urban geographies while being apparently too big to control, too new to regulate, and too innovative to stifle*”, this paper argues for three major interdependent reasons as to why it is difficult to pinpoint digital platforms embeddedness.

### Unawareness

Platforms often remain unacknowledged as such, since many processes are neither directly visible nor perceived as platform-related. This invisibility applies not only to products and services but also often to the responsible companies as well as their employees and consequently to ‘space’ itself. While classical production plants or traditional shops can be assigned to a fixed location and thus a relationship to the physical environment of the companies is created, the platform economy takes place largely in a virtual environment, often within our own four walls. This resides not only in the fact that we blithely take digital platforms in our bedrooms in form of our smartphones or that a representative of the platform ‘sits’ with us at the dining table in the shape of Siri and Alexa, while we are agreeing to their terms and conditions even in our most private spaces. This also includes the fact, that platforms create new markets with novel ways of generating value by establishing new commodities. This includes private cars and houses, turning citizens into entrepreneurs by offering their homes and automobiles (Törnberg and Chiappini 2020) as well as the personal data of customers, turning the customers into products themselves (Barns 2019). Thereby gamification, the application of typical elements of game playing (e.g. point scoring, competition with others, rules of play) to other areas of activity, typically as an online marketing technique to encourage engagement with a product or service, plays an essential role. It incentivizes the production of ever more relational data to further advance the very models of social behavior on which gamification is based, as revealed by the examples of LinkedIn and Facebook (Cohen 2020). To increase the completion rate, LinkedIn uses a simple but effective tool: the progress bar. After successfully entering all relevant data, members also receive a badge - they are now ‘superstars’. The role of the user is no longer simply to use but to offer user feedback and produce data for the digital platforms (Easterling 2016; Grabher and König 2020), converting transformation of daily routines into laboratory sites for product and service development. This manifests itself in a hierarchical relationship between users and digital platforms, leading to an extreme power asymmetry in which the platform clearly controls the nexus of relationships (Cutolo and Kenney, 2019). The ability of platform companies to assume dominant market positions and systematically change economic structures depends to a large extent on this power asymmetry, which also finds its roots in their ability to circumvent regulations (Graham 2020; Katta et al. 2020).

### Unaccountability

As it is reasoned that digital platforms hold the power to create their own logic (Frenken and Schor 2017; Schwarz 2017), they can also influence the logics of other institutions, such as the state and the market. Yet, despite digital platform’s perception as ‘governing systems’ that *“control, interact, and accumulate*” (e.g. Schwarz 2017: 1), they escape accountability due to their “*un-democratic, and usually distant*” character, revealing “*no interest in promoting local voices or investing in local priorities*” (Graham 2020: 2). That can be attributed to one underlying characteristic of digital platforms, often decoupling themselves from the commodities they sell / channel. Uber and Airbnb for instance label themselves as technology companies instead of transportation or accommodation companies, respectively not owning a single vehicle or hotel room, claiming that they are simply a marketplace. Further, Uber classifies drivers as ‘independent contractors’ rather than ‘employees’, therewith avoiding labour protections such as sick pay or minimum wages (Wood et al. 2019; Chen and Sun 2020; Walker 2020). Every single Uber driver has a contract with Uber International Holding(s) BV (a Netherlands-based company). In this way – “*by operating at a different spatial scale to their workers”* (Graham 2020: 3) and avoiding local responsibility, digital platforms remain unaccountable (Huws (2016) 1) refers to these circumstances as the “*virtual wild west*”). A further liability evasion derives from what (Grabher and König (2020) 108) label the “*key arena of regulatory struggles*”, which is defined by a vast amount of Terms-of-Use agreements (Zuboff (2019) 220) calls them “*uncontracts*”). These invoke the notion of a public domain that enables legal privileges, while being “*performative acts of consummation*”, as data is converted “*into de facto property arrangements*” (Cohen 2019: 242), mobilizing an altered understanding of legality (Cohen 2020: 3). Further, as pointed out by (Katta et al. (2020) 2), digital platforms disembed themselves “*from the tax jurisdictions of the localities they serve, leaving treasuries short-changed*.”

### Non-transparency

In contrast to the unawareness, non-transparency refers to the secrecy in which digital platforms operate internally. The interplay of algorithms and venture capital offices provides one possible reason for the lack and difficulty of conducting studies on digital platforms. They may be perceived as black boxes *“due to the proprietary nature of algorithms, the secrecy of corporate ownership structures, and the emphasis on confidentiality and privacy in the venture capital industry*” (Fields et al. 2020: 462). Usually, there is no office door to knock at, as platforms may not have any physical presence in the cities they operate. Further, as in the case of Google or Facebook, there is no phone number to ring, something most consumers would expect to be able to get in touch. Yet, they allow users some access by agreeing to a set of terms and conditions in order to enter “*private property*” (Kenney et al. 2019: 3), reserving nearly total control to the digital platform, the owner respectively, which is also enhancing the unaccountability of the platforms, as they are left “*with little or no public oversight*” (Kenney et al. 2019: 2). This is in particular true as platforms can change their algorithms, e.g. a transaction platform can modify its recommendation algorithms to put more emphasis on price or search engines’ algorithms that determine what one sees when a search query is typed into them.

Importantly, the three factors are interdependently, they overlap, influence and mutually reinforce each other. They all lead to the fact that platforms evolve around ambivalent, even contradictory processes. (Fields et al. (2020) 465) argue, “*that a focus on the apparent opacity of platforms may reify them as external to, rather than thoroughly embedded in, the relations among devices, people, and the urban.”* Yet, while they *disembed [ded] from the space-times they mediate*” (Graham 2020: 1), they simultaneously are embedded in daily routines and the urban fabric - in some circumstances to an almost unknown degree. This “*strategic deployment of ‘conjunctural geographies’*” (Graham 2020: 1) allows platforms to hold an “*instrumentarian power*” to modify and monetize social behavior (Zuboff 2019: 139). Further, they even “*co-produce their own institutional and societal embeddedness*” (Grabher and van Tuijl 2020: 7). Indeed, this frontier paper argues that the (dis-)embedding processes are becoming even more complex, as the roles of different actors are increasingly blurred and their interactions are less spatially relevant.

## (dis-)embeddedness and its usefulness for future research

Recently, research in the digital platform economy has started to focus on embeddedness aspects (e. g. Shevchuk and Strebkov 2018; Fan et al. 2019; Montalban et al. 2019; Graham 2020; Grabher and König 2020; Kenney et al. 2020). These studies have found the concept of embeddedness valuable, although they have mainly concentrated around the issues of labour in the platform economy. Fan et al. (2019) examined the concept of societal embeddedness by investigating the influence of social embeddedness on organizational legitimacy and the sustainability of the globalization of the sharing economic platform, using the example of Uber China. Wood et al. (2019) explore the (dis) embeddedness of digital labour within the remote gig economy in Southeast Asia and Sub-Saharan Africa and conclude that normative disembeddedness leaves workers exposed to the vagaries of the external labour market due to an absence of labour regulations and rights, while simultaneously being embedded within interpersonal networks of trust. Similarly, (Shevchuk and Strebkov (2018) 1) investigate inter alia the social embeddedness of more than 5000 Russian-speaking freelancers from an international online labour market. Importantly, Wood et al. (2019) as well as Grabher and König (2020) emphasize the importance of ‘hard’ Polanyi, the commodification aspect respectively – a component often ignored when using Polanyi’s analysis of what he termed the ‘Great Transformation’ to frame the rise of the platform economy, confirming the accompanying increasing commodification. (Wood et al. (2019) 5) term the “*alternative hard-Polanyi form of societal embeddedness*” ‘normative embeddedness’ in order to distinguish it from extant uses. Indeed, they (2019: 16) call for commodification to be in the centre of societal (dis) embeddedness, while seeing prospective outcomes of Polanyis ‘double movement’. They (2019: 14) find that “despite labour remaining embedded within workers’ interpersonal networks, it is at the same time being disembedded from cultural and legal norms that would limit its commodification.” Montalban et al. (2019) critically ask whether platform economies represent a new process of embeddedness or a next step for deregulation following the crisis of the financialised regime of accumulation, which consequently may favour some forms of dis- or re-embeddedness. Dis-embedding processes are also described by Graham (2020) and Katta et al. (2020) regarding Uber and labour. Graham (2020) 4) argues that the sole reliance on the mediation of data and information (owning no taxis as in the case of Uber, or housing as in the case of Airbnb), makes digital platforms “*vulnerable to local alternatives*”, consequently, calling for avoidance, replication, regulation as well resistance. Katta et al. (2020) discusses an interesting example: Due to the Covid-19 pandemic and the associated lockdowns, most Uber drivers were left without a viable income. Due to public pressure however, Uber offered, “*modest employment benefits, including limited paid sick leave*” (Katta et al. 2020: 2). In California for instance “*the state’s recent AB5 law (that would see gig workers receive employee status) presents an existential threat to the company’s business model ( …) implicitly acknowledge that Uber [is] far more than the proprietor of a digital marketplace*” (Katta et al. 2020: 3). This reveals that regulations and policies can have a tremendous influence on the embeddedness as well as the actual existence of the respective digital platform. Another example represents Amazon in India, where the government ruled that simultaneously being a platform for third-party sellers and a vendor itself is impossible (BBC 2019). These examples confirm Graham’s (2020) argument, that digital platforms are vulnerable, “*enabling that embeddedness provides a pathway to encoding local accountability into the gig economy’s script*.” Indeed, this might be (to offer an optimistic outlook) the start of what could be observed in the aftermath of the industrial revolution, when labour via the establishment of trade unions and other institutional involvements was gradually ‘de-commodified’ (although this development has been reversed – under the term ‘re-commodification’ since the 1980s) (Wood et al. 2019: 6). This draws parallels to Stehlin et al.’s (2020) 14) argumentation that due to platforms spatial embeddedness, they cannot simply wipe out the “*localized configurations of political economic power, territorial authority, infrastructural history, and socio-spatial inequality [ …] and begin with a tabula rasa.*”

Therefore, geographic research on platform urbanism must deepen our understanding on how platforms are situated within the blurring interconnections and actors of the city. The concept of embeddedness serves as a valuable tool to understand and pinpoint essential dynamics and relationships of the platform economy, broadening our understanding of the underlying powers and arenas as well as participants that play a crucial role in the platform economy, therewith overcoming the unawareness, unaccountability and non-transparency towards and of digital platforms. Further, without denying the importance of current research focusing on what might be termed ‘new traditional’ labour (e.g. Uber driver), this paper argues that the commodification of seemingly irrelevant ‘labour’ by consumers should also move in the centre of attention, as the borders of home, leisure, consumption and work blur, contributing to the unawareness of digital platforms power and reach. A full (er) picture of the embeddedness and the respective dis-embeddedness processes of platform companies has to be gained in order to proceed with the shaping of the platform economy which currently represents “*an inevitable urban future of capitalism stripped down to its essentials*” (Graham 2020: 4). (Kenney et al.’s (2019) 8) posing of two fundamental questions: “*How embedded are digital platforms in the economy, and, conversely, how embedded is the economy in digital platforms?*” are essential, but not far-reaching enough. We have to broaden the concept of embeddedness, especially due to enormous power of platforms to (re-)create large parts of our urban and societal world. Therefore, I follow Peck’s (2013) plea “*for a more sustained - but also open, critical, and creative - engagement with Polanyi’s legacy.*” Deriving mainly from the three factors detailed above (unawareness, unaccountability and non-transparency), a variety of research questions and suggestions are apparent of which few should be named here: Which implications flow from the changing role of consumers/users on the (dis-)embeddedness of digital platforms? How can the increasing amalgamation of home, leisure, consumption and work be translated into embeddedness. How can we distinguish them and is it necessary to distinguish them? How does (dis-)embeddedness differ between large/small platforms and what roles do different sectors play? What role does the cultural backgrounds of decision-makers within the platforms play? How does accountability influence the embeddedness of digital platforms and vice versa? And on a more general basis, as pointed out at the beginning, definitions and categorization of platforms vary and sometimes even contradict each other. Might the embeddedness (or dis-embeddedness) itself represent a good foundation for the categorization of digital platforms? Generally, studies such as Wood et al. (2019) as well as Graham (2020) clearly demonstrate the value of an integrated understanding of Polanyi’s theorization of embeddedness for analysing contemporary economic transformations. Embeddedness might help us rethink the concept of digital platforms as fluid, multi-layered companies that have no fixed spatial location outside of their corporate headquarters, who have an ability to link “*themselves to the local to concentrating reward, and retreat to their ephemeral digital dualisms when abdicating responsibility*” (Graham 2020: 2). It is argued that a rigorously identification and examination of the ‘layers’ of embeddedness is necessary, thereby re-evaluating the questions: embeddedness of what, to what, by what means, and with what outcome?

## Data Availability

Not applicable.
